# A Giant Extracellular Matrix Binding Protein of *Staphylococcus epidermidis* Binds Surface-Immobilized Fibronectin via a Novel Mechanism

**DOI:** 10.1128/mBio.01612-20

**Published:** 2020-10-20

**Authors:** Henning Büttner, Markus Perbandt, Thomas Kohler, Alexey Kikhney, Manuel Wolters, Martin Christner, Marisol Heise, Jérôme Wilde, Samira Weißelberg, Anna Both, Christian Betzel, Sven Hammerschmidt, Dmitri Svergun, Martin Aepfelbacher, Holger Rohde

**Affiliations:** a Institut for Medical Microbiology, Virology, and Hygiene, University Medical Center Hamburg-Eppendorf, Hamburg-Eppendorf, Germany; b Department of Molecular Genetics and Infection Biology, Interfaculty Institute for Genetic and Functional Genomics, Center for Functional Genomics of Microbes, Greifswald, Germany; c European Molecular Biology Laboratory, Hamburg Unit, Hamburg, Germany; d University of Hamburg, Laboratory for Structural Biology of Infection and Inflammation, Hamburg, Germany; Institut Pasteur

**Keywords:** *Staphylococcus*, biofilms, fibronectin binding, surface proteins, surface structures

## Abstract

Although it is normally an innocuous part of the human skin microbiota, Staphylococcus epidermidis has emerged as a major nosocomial pathogen, and implanted foreign materials are an essential risk factor for the development of an infection. The extraordinary efficiency of S. epidermidis to colonize artificial surfaces is particularly related to the ability to form biofilms. Biofilm formation itself critically depends on stable pathogen binding to extracellular host matrix components, e.g. fibronectin (Fn), covering inserted devices in vast amounts. Extracellular matrix binding protein (Embp) and its subdomains referred to as the F-repeat and the FG-repeat are critical for adherence of S. epidermidis to surface-immobilized Fn. Embp-Fn interactions preferentially occur with surface-bound, but not folded, globular Fn via binding to the F3 domain. High-resolution structure analysis of F- and FG-repeats revealed that both repeats are composed of two tightly connected triple α-helix bundles, exhibiting an elongated but rather rigid structural organization in solution. Both F- and FG-repeat possess Fn-binding capacity via interactions with type III subdomain FN12, involving residues within the C and F β-sheet. FN12 essentially supports stability of the globular Fn state, and thus these findings reasonably explain why Embp-mediated interaction of S. epidermidis necessitates Fn surface immobilization. Thus, Embp employs an uncharacterized bacterial Fn-binding mechanism to promote staphylococcal adherence.

## Abstract (precis)

Staphylococcus epidermidis is a leading pathogen in implant-associated hospital infections. The pathogenesis critically depends on bacterial binding to ECM components, specifically fibronectin (Fn). The cell surface-localized, 1-MDa extracellular matrix binding protein (Embp) is essentially characterized by 10 F- and 40 FG-repeats. These repetitive units, each characterized by two α-helical bundles, organize themselves in a rigid, elongated form. Embp binds preferentially to surface-localized but not soluble Fn, with both F- and FG-repeats being sufficient for Fn binding and resulting bacterial adherence. Binding preferentially involves Fn type III domain, specifically residues of FN12 β-sheets C and F. Both play key role in stabilizing the globular Fn conformation, explaining the necessity of Fn surface immobilization for a subsequent interaction with Embp. In comparison to many other bacterial Fn-binding proteins using the Fn N terminus, Embp employs a previously undescribed mechanism supporting the adhesion of S. epidermidis to surface-immobilized Fn.

## INTRODUCTION

Staphylococcus epidermidis is a leading cause of health care-associated infections (1, 2). Infections typically occur after implantation of medical devices, e.g., central venous catheters, prosthetic heart valves, or prosthetic joints. Being usually considered a harmless skin commensal, the selective pathogenic potential of S. epidermidis in foreign-material associated infections is related to the pronounced biofilm forming ability of this species (3). Biofilm formation protects S. epidermidis from effectors of the host immune system and induces phenotypic resistance even against antimicrobial agents that have been tested susceptible under standard laboratory conditions (4–6). As a clinical consequence, these properties result in chronic, hard-to-treat infections.

**FIG 1:**
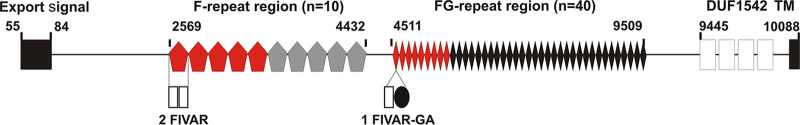
Schematic representation of the Embp architecture. The 1-MDa Embp carries two major repetitive regions consisting of repeats each encompassing 170 and 125 amino acids (aa), respectively. The 170-aa repeat is referred to as F-repeat and is present in 10 copies (indicated by pentagons). The 125-aa repeat, referred to as FG-repeat, can be found in 40 copies (each indicated by a diamond). Previous bioinformatics analysis (22) identified 22 **F**ound **i**n **V**arious **Ar**chitectures (FIVAR) modules within the F-repeat region (indicated by open boxes). One F-repeat is represented by two FIVAR modules. The FG-repeat region is predicted to contain 38 **G**-related **A**lbumine binding (GA) modules (indicated by a filled circle), each associated with one FIVAR module. Each FG-repeat represents a pair of GA and FIVAR modules. Experimental evidence demonstrates that the predicted modular architecture does not match the actual structural organization derived from X-ray crystallography (Fig. 5). An N-terminal export signal containing an YSIRK motif, C-terminal domains of unknown function (DUF1542) and a putative transmembrane region (TM) are bioinformatics predictions. Pentagons and diamonds filled red indicate five F-repeats and nine FG-repeats that were fused to the export signal and the putative cell wall binding region for in *trans* expression in staphylococci. Upper numbers indicate amino acid positions referring to the Embp amino acid sequence.

Molecular work from the past decades revealed that S. epidermidis biofilm formation is a multistep process involving a plethora of bacterial molecules, all integrated into complex regulatory circuits (7, 8). Many of these factors are well-characterized adhesins (e.g., polysaccharide intercellular adhesin [PIA], accumulation associated protein [Aap], extracellular DNA [eDNA] [9]) fostering intercellular adhesion, cell aggregation and, ultimately, the establishment of a multicellular biofilm architecture (10). Although aggregation is a key aspect of S. epidermidis biofilm formation, the stable attachment of multicellular biofilm aggregates to the implant surface represents the critical step during successful colonization of a foreign material. S. epidermidis attachment to artificial surfaces is fostered by a number of bacterial factors, including specific interactions between the bacterial cell surface proteins (e.g., major autolysine E, AtlE [11], and Aap [12]) and the foreign materials. Importantly, after introduction of foreign material into the human body, medical devices are almost immediately covered (“conditioned”) by serum proteins and host-derived extracellular matrix (ECM) components, e.g., fibronectin (Fn), fibrinogen, vitronectin, and thrombospondin (13–15). In turn, bacterial cell surface proteins specifically binding serum and ECM components (referred to as microbial surface components recognizing adhesive matrix molecules [MSCRAMM] [16]) are of critical importance for the initiation of surface colonization. In fact, S. epidermidis produces several MSCRAMMs that specifically interact with fibrinogen (i.e., SdrG) (17), collagen (i.e., GehD) (18), vitronectin, and thrombospondin (i.e., AtlE) (11, 19). Along with teichoic acids (20), mechanisms supporting S. epidermidis binding to surface organized Fn, however, remained obscure. The extracellular matrix binding protein (Embp) is among the first dedicated S. epidermidis Fn-binding cell surface proteins involved in bacterial adhesion to surface-immobilized Fn (21, 22). Embp is a giant 1-MDa surface protein consisting of 10,203 amino acids (aa). According to bioinformatics primary structure analysis Embp contains an N-terminal export signal, followed by an unstructured region having been implicated in osmoresistance in the S. aureus Embp homolog Ebh (23). The immediate neighboring region is characterized by 21 Found in various architecture (FIVAR) elements, followed by 38 repetitive units in which each FIVAR element is associated with one G-related albumin binding module (GA). At the C terminus, four domains of unknown function (DUF1542) and a putative transmembrane region are predicted (22, 24) (Fig. 1).

The major goal of the present study was to characterize molecular determinants of Embp-Fn interactions and to elucidate the functional relevance for S. epidermidis adherence to surface-immobilized Fn. Crystal structure analysis of core Embp units and their functional characterization provide novel insights into a unique bacterial mechanism contributing to Fn binding and surface colonization.

## RESULTS

### Embp is crucial for *S. epidermidis* interactions with fibronectin.

The ECM binding protein Embp possesses Fn-binding activity (21, 22). Aiming at elucidating the molecular basis of the Embp-Fn interaction, experiments were carried out in which binding of staphylococci expressing Embp, defined Embp fragments, or S. aureus Fibronectin binding Protein A (FnBPA) to soluble or immobilized Fn, as well as to Fn subdomains, were tested. FnBPA served as a control due to its well characterized binding activity to N-terminal type I Fn modules (25).

**FIG 2:**
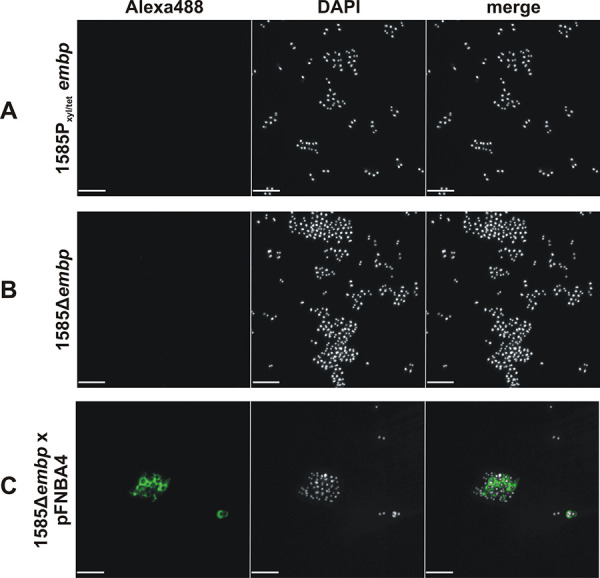
Recruitment of soluble fibronectin to Embp- or FnBPA-expressing S. epidermidis. S. epidermidis 1585Δ*embp*, 1585Δ*embp* × pFNBA4, and 1585*P*_xyl/tet_*embp* were incubated with fibronectin. After washing, cell surface-localized Fn was detected using rabbit anti-Fn IgG and Alexa 488-conjugated anti-rabbit IgG. Bacteria were stained with DAPI (white) and Fn (green). While S. epidermidis 1585Δ*embp* × pFNBA4 is able to recruit soluble Fn to the cell surface indicated by green fluorescence signal (C), no Fn is detected on the surface of Embp expressing 1585*P*_xyl/tet_*embp* (A); S. epidermidis 1585Δ*embp* served as a negative control (B). Scale bar, 5 μm.

**FIG 3:**
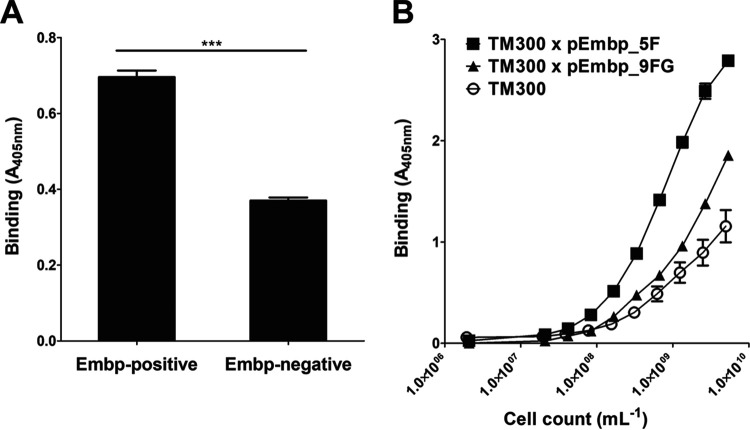
Adherence of Embp-producing staphylococci to full-length Fn. A 96-well microtiter plate coated with 10-μg/ml full-length Fn was incubated for 1 h with staphylococci. After washing, bacteria were indirectly quantified by a colorimetric reaction. (A) Comparison of the Fn adherence of S. epidermidis 1585 and its isogenic *embp* deletion mutant grown under *embp* inducing conditions (50) shows a clear reduction in the bacterial load, although background binding is still observed that might be attributed to Embp-independent cell surface structures. (B) Binding of *S. carnosus* TM300 × pEmbp_5F and *S. carnosus* TM300 × pEmbp_9FG (expressing five F- or nine FG-repeats, respectively) to surface-immobilized Fn. Both, F- and FG-repeats support binding to Fn. Wild-type *S. carnosus* TM300 served as a control. ***, *P* < 0.0001 (Student *t* test).

In order to test the effect of Embp production on cell surface recruitment of soluble Fn, S. epidermidis 1585*P*_xyl_*_/_*_tet_*embp*, a strain in which the *embp* expression can be specifically switched on and off via a tetracycline inducible promoter (22), and the *embp* knockout mutant S. epidermidis 1585Δ*embp* was suspended in phosphate-buffered saline (PBS) containing Fn (10 μg/ml) for 1 h. After subsequent washing, an anti-Fn monoclonal antibody was unable to detect significant amounts of Fn on the surface of *embp*-induced S. epidermidis 1585*P*_xyl/tet_*embp* (Fig. 2A), resembling the finding made with the *embp* knockout mutant S. epidermidis 1585*Δembp* (Fig. 2B). In sharp contrast, in *trans* expression of FnBPA in *embp* knockout mutant S. epidermidis 1585Δ*embp* leads to a strong Fn surface decoration (Fig. 2C). In adherence assays, however, Embp-producing S. epidermidis 1585*P*_xyl/tet_*embp* more efficiently bound to surface deposited Fn compared to the uninduced control (Fig. 3A). Collectively, these data indicate that full-length Embp interacts with surface-immobilized Fn and fosters S. epidermidis adherence to Fn-coated surfaces, while the protein is unable to bind to and recruit soluble Fn. Thus, regarding interactions with Fn, these findings suggest that Embp possess significant functional differences compared to S. aureus FnBPA.

### Embp is sufficient for the adherence of staphylococci to surface-immobilized Fn.

Previous work has shown that Embp is essentially characterized by two repeating regions. The N-terminal repetitive region (aa 2569 to 4432) consists of 10 repeats encompassing 170 aa (Fig. 1). These repeats are identified by several highly conserved amino acids (see Fig. S1A in the supplemental material) and are here referred to as the F-repeat. The C-terminal repetitive region (aa 4511 to 9509; Fig. 1), also present in S. aureus homolog Ebh, is composed by a row of 40 repeats consisting of 125 aa. This repeating unit (see Fig. S1) is referred to here as the FG-repeat. To test whether F- or FG-repeats independently foster S. epidermidis adherence to Fn, constructs pEmbp_5F and pEmbp_9FG, allowing for in *trans* expression of five F- or nine FG-repeats (Fig. 1), fused to the predicted cell wall anchor region (i.e., DUF1542 modules and the transmembrane domain) were used to transform *S. carnosus* TM300. *S. carnosus* TM300 expressing five F- or nine FG-repeats, respectively, more efficiently adhere to surface immobilized Fn compared to Embp-negative TM300 (Fig. 3B). Thus, both F- and FG-repeats are obviously independently sufficient for adherence to Fn.

10.1128/mBio.01612-20.1 FIG S1 PROMALS3D alignment of 10 F- and 40 FG-repeats. (A) Alignment of predicted 10 F-repeats using first F-repeat (rF-repeat; Fig. 3A) as a structure template. An overall agreement for the positioning of secondary structure elements of the F-repeat structure and the PROMALS3D alignment (cons_ss) is indicated. (B) Alignment of predicted 40 FG-repeats (including r-FG-repeat expressed for structure elucidation, first line) using EbhA-R7-R8 (29) as a structure template (second line). Amino acid positions 66 to 193 of EbhA-R7-R8 align with the predicted FG-repeats. In addition to amino acid homology also secondary structure elements in EbhA-R7-R8 match the PROMALS3D predictions (cons_ss). Cons_ss, conserved secondary structure features; h:, helix. Download FIG S1, TIF file, 2.7 MB. Copyright © 2020 Büttner et al. 2020 Büttner et al. This content is distributed under the terms of the Creative Commons Attribution 4.0 International license.

**FIG 4:**
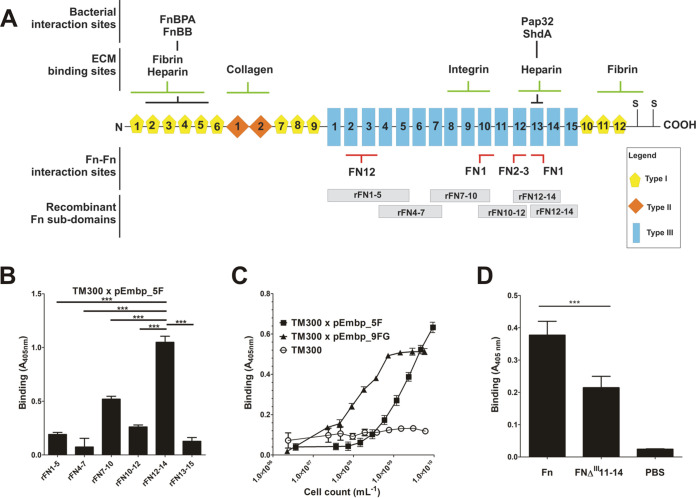
(A) Schematic representation of the cellular fibronectin monomer. Fibronectin is a 250-kDa multidomain glycoprotein found in various body fluids and various tissues. The type I domain (yellow pentagons) consist of 12 repeats of about 40 aa. Repeats F1_6_ and F1_7_ are intersected by two type II repeats (F2_1_ and F2_2_, each consisting of 60 aa; orange rhomboid), forming the type II domain. In total, at least 15 Fn type III repeats (each consisting of 90 aa) form the type III domain. The secondary structure of type I and type II repeats are stabilized by disulfide bonds. Their absence in type III repeats is related to the elasticity and plasticity of the Fn type III domain. A globular Fn conformation is stabilized by several Fn domain interactions between the two strands of the Fn dimer (e.g., FN12–FN2-3, FN1–FN10, and FN1–FN13; indicated by red bars). Interaction sites with bacterial adhesins (FnBPA [S. aureus], FnBB [S. dysgalactiae], Pap32 [B. henselae], ShdA [S. enterica]), or host extracellular matrix components are indicated by black and green bars, respectively. The positions of recombinant Fn subdomains are indicated by gray boxes. S, position of cysteine residues involved in covalent Fn dimer formation. Extra domains A and B and a variable domain present in plasma Fn are not shown. The figure was adapted from Kubow et al. (46). (B) Adherence of *S. carnosus* expressing five F-repeats to overlapping Fn type III subdomains. Recombinant Fn type III subdomains rFN7-10, rFN4-7, rFN7-10, rFN10-12, rFN12-14, and rFN13-15 (see panel A) were immobilized on an Immobilizer microtiter plate and incubated with TM300 × pEmbp_5F (10^8^/ml) for 1 h. The unmodified surface served as a control. Adherent bacteria were detected using a polyclonal rabbit anti-S. epidermidis serum and alkaline phosphatase-conjugated anti-rabbit IgG. Bars represent bacterial binding (expressed as the absorption at 405 nm) after background subtraction. Differences between binding to rFN12-14 were significantly different compared to all other recombinant Fn fragments tested (*P* < 0.0001, Student *t* test). (C) Adherence of *S. carnosus* × pEmbp_5F and *S. carnosus* × pEmbp_9FG to surface-immobilized rFN12-14. A 96-well Immobilizer microtiter plate surface (Nunc, Roskilde, Denmark) was coated with recombinant fibronectin subdomains. Increasing numbers of *S. carnosus* TM300 × pEmbp_5F and *S. carnosus* TM300 × pEmbp_9FG were incubated for 1 h on the surface. Adherent bacteria were detected using a polyclonal rabbit anti-S. epidermidis serum and alkaline phosphatase-conjugated anti-rabbit IgG. *S. carnosus* TM300 wild type served as a control. (D) Adherence of S. epidermidis 1585P_xyl/tet_*embp* to Fn and FnΔ^III^11-14. Full-length Fn and an isoform lacking type III subdomains FN11 to FN14 (FnΔ**^III^**11-14) were purified from supernatants of HEK293 cells transiently transfected with FN-YPet/pHLSec2 or FNΔ^III^11–14/pHLSec2. Fn isoforms were immobilized on a microtiter plate and incubated with S. epidermidis 1585P_xyl/tet_*embp* grown under *embp*-inducing conditions. After washing, adherent bacteria were detected using a polyclonal rabbit anti-S. epidermidis serum and alkaline phosphatase conjugated anti-rabbit IgG antibody. ***, significant (*P* < 0.0001) difference (Student *t* test).

The glycoprotein Fn is a multidomain protein found in various body fluids and tissues. It is mainly composed of three distinct domains, referred to as type I, type II, and type III domains (Fig. 4A). Previous work using far-Western blotting demonstrated that Embp interacts with C-terminal Fn regions (26), notably type III repeats 12 to 14 (22). To more precisely describe the relevance of this finding for S. epidermidis-Fn interactions, Embp-mediated adherence to defined Fn subdomains was evaluated. To this end, overlapping recombinant Fn fragments covering Fn type III subdomains 1 to 15 (27) were expressed as His_6_ fusion proteins and bound to Immobilizer microtiter plates, dedicated for immobilization of His tag fusion proteins. Coated plates were used in adherence assays using *S. carnosus* TM300 carrying pEmbp_5F. Interestingly, bacterial adherence was primarily supported by rFN7-10 and rFN10-12, whereas other recombinant F3 subdomains only weakly increased bacterial binding, and rFN12-14, the latter showing the most pronounced binding (Fig. 4B). Given the pronounced binding to rFN12-14, this fragment was chosen further comparative functional analysis focused on interactions of F- or FG-repeats. Compared to the wild type, *S. carnosus* TM300 expressing either F- or FG-repeats showed strongly increased adherence to microtiter plates coated with rFN12-14, thus demonstrating that either region is sufficient to foster bacterial adherence to rFN12-14 (Fig. 4C). Of note, *S. carnosus* TM300 expressing nine FG-repeats more efficiently supported bacterial adherence to rFN12-14 in comparison to expression of five F-repeats (Fig. 4C).

In order to more precisely clarify the significance of FN12 for the Embp-mediated adherence of S. epidermidis to Fn, a FN12-deficient Fn isoform (FNΔ**^III^**11-14) (28) was expressed in HEK293 cells, purified via gelatin agarose chromatography and immobilized on microtiter plates. In parallel, full-length wild-type Fn was produced, purified, and immobilized using an identical procedure. Embp-producing S. epidermidis 1585*P*_xyl/tet_*embp* was then allowed to adhere to FNΔ**^III^**11-14 or Fn conditioned surfaces. Compared to the uncoated control surface, and in line with experiments using commercial Fn preparations, coating the surface with purified wild-type Fn led to a strong increase in the adherence of S. epidermidis 1585*P*_xyl/tet_*embp* (Fig. 4D). Compared to wild-type Fn, deletion of FN11-14 in FNΔ^III^11-14 resulted in a clear 43% reduction of bacterial adherence (Fig. 4D). Although residual binding was still evident, this finding supports the assumption that, indeed, type III repeats, including FN12, are functionally important for Embp-mediated S. epidermidis adherence to surface-localized Fn.

### Embp F- and FG-repeats consist of two interconnected triple α-helix bundles.

**FIG 5:**
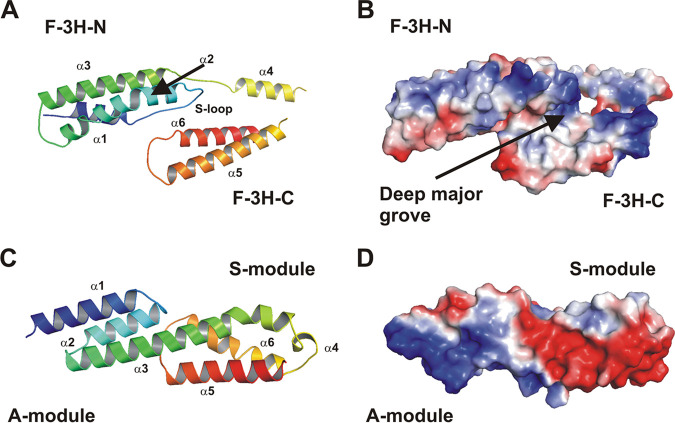
Structural analysis of Embp. (A) Cartoon plot of the F-repeat showing the three helix-bundle arrangement. N-terminal helix bundle F-3H-N (aa 2569 to 2652) and C-terminal helix bundle F-3H-C (aa 2660–2738) are connected by a short linear linker (L2, aa 2653 to 2659). Within F-3H-N, helix α1 and α2 are connected by a Sandwich-loop (S-loop) which potentially is of fundamental importance for the structural integrity of the F-repeat as a compact unit. Helix α2 shows a remarkable almost 45° kink at residue Ala-2607. The third helix α3 (aa 2629 to 2652) follows in the opposite direction after a short loop (L1, aa 2624 to 2628). Helix α3 is slightly bended to allow a tight interaction between all three helices. Within F-3H-C, the first helix α4 (aa 2660 to 2673) is followed by the second helix α5 (aa 2685 to 2709). The connecting loop L3 (aa 2674 to 2684) is distorted and not defined by the electron density. Helix α5 (aa 2685 to 2709) and helix α6 (aa 2720 to 2738) are connected by loop L4 (aa 2710 to 2719). The model is colored according to the sequence, from blue at the N terminus to red at the C terminus. The other structural elements are assigned accordingly. (B) An electrostatic surface potential representation in the same orientation as in panel A reveals a prominent deep major groove flanked by helix α2 (from F-3H-N) and helix α4, α5, and α6 (from F-3H-C). (C) Cartoon plot of the FG-repeat showing the three helix-bundle arrangement composed of an A- and S-module. At the junction between the A- and S-module, the completely conserved residues, Leu-6798, Gln-6802, and Leu-6829 (from A-module), create a hydrophobic core with highly conserved hydrophobic residues, Met-6833, Ile-6882, and Ile-6887 (from S-module), which are further stabilized by a direct hydrogen bond between Gln-6802 and Ile-6882. Therefore, both three-helix bundles of the FG-repeat are connected rather tightly. Helix α4 is running perpendicular to all other helices and connects helix α3 and helix α5. These structural features are consistent with those described for EbhA-R7-R8 (29) from S. aureus Ebh. The model is colored according to the sequence, from blue at the N terminus to red at the C terminus. The central long helix α3 connects the A- and S-module and is colored in green. (D) An electrostatic surface potential representation in the same orientation as in panel C reveals a remarkable strong dipole character of the FG-repeat. The N-terminal region of the A-module is strongly positively charged, whereas the C-terminal region (S-module) is strongly negatively charged.

In order to gain insights into the structural basis of Embp-mediated S. epidermidis Fn binding, experiments were initiated aiming at resolving the structure of Embp regions possessing Fn-binding activity. A high-resolution crystal structure was obtained for the first F-repeat (corresponding to aa 2569 to 2738 of the full-length Embp). The structure was determined by single-wavelength anomalous diffraction (SAD) method using data from the Se-Met-variant that was refined at a 1.39-Å resolution using high-resolution diffraction data of the native F-repeat (PDB entry code 6GV8). The F-repeat consists of two three-helix bundles and reveals an elongated shape approximately 70 Å in length and 30 Å in width and height (Fig. 5A). The N-terminal bundle is referred to as F-3H-N, and the C-terminal bundle as F-3H-C. Within F-3H-N, the first helix α1 (aa 2571 to 2584) and the second helix α2 (aa 2599 to 2623) are connected by an extended loop (aa 2585 to 2598). This loop (referred to as the Sandwich-loop [S-loop]) is involved in several stabilizing hydrogen bonds and van der Waals interactions between F-3H-N, a linear linker L2 and F-3H-C (Fig. S2), potentially important for the structural integrity of the F-repeat as a compact unit.

10.1128/mBio.01612-20.2 FIG S2 The Sandwich-loop (S-loop, residues 2585 to 2598) reveals several important stabilizing hydrogen bonds and van der Waals interactions between F-3H-N, the linear linker L2, and F-3H-C. Download FIG S2, TIF file, 0.8 MB. Copyright © 2020 Büttner et al. 2020 Büttner et al. This content is distributed under the terms of the Creative Commons Attribution 4.0 International license.

The analysis of the F-repeat surface characteristics reveals a prominent deep major groove as key structural feature (Fig. 5B). The wide groove shows an area of approximately 1,200 Å^2^ comprising a volume of 890 Å^3^ and is flanked by helix α2 (from F-3H-N) and helices α4, α5, and α6 (from F-3H-C). Considering its size, this groove may serve as a potential binding pocket for ligands. Intriguingly, despite low overall sequence homology within the 10 F-repeats, secondary structure predicts a highly conserved α-helical organization similar to the first F-repeat (see Fig. S1A).

The crystal structure of the 19th FG-repeat (aa 6777 to 6902) was determined by molecular replacement (MR) to a resolution of 1.55 Å (PDB entry code 6GV5). The repeat consists of two three-helix bundles, referred to as the A-module and the S-module (Fig. 5C; Fig. S3), and despite the low sequence identity of 30%, the overall structure is in good agreement with the corresponding substructure of two repeats R7-R8 from the S. aureus Embp homologue Ebh (PDB entry code 2DGJ [29]). Both corresponding regions can be superimposed with a root mean square deviation (RMSD) of 1.6 Å for corresponding atoms (see Fig. S3). Similar to the F-repeat, the FG-repeat reveals an elongated shape approximately 70 Å in length and 20 Å in width and height. A- and S-modules are connected by a shared helix α3, lancing through both three-helix bundles (Fig. 5D; the length of the central helix α3 is about 40 Å).

10.1128/mBio.01612-20.3 FIG S3 Superposition of the FG-repeat homology model (colored) and the crystal structure of EbhA-R7-R8 (gray). The overlap covers EbhA-R7-R8 amino acids 73 to 198. The coverage of the aligned sequence is 99.2%. As quality assessment for the match, a template modeling (TM) score of 0.984 and RMSD of 0.37 **Å** indicate the validity of the alignment. Download FIG S3, TIF file, 0.4 MB. Copyright © 2020 Büttner et al. 2020 Büttner et al. This content is distributed under the terms of the Creative Commons Attribution 4.0 International license.

The FG-repeat is characterized by a remarkable strong dipole character (Fig. 5D). The N-terminal region (A-module), formed by the N terminus of helix α1 and the N terminus of helix α3, is strongly positively charged, whereas the C-terminal region (S-module) formed by the C terminus of helix α6 and the short helix α4, is strongly negatively charged. Consistent with F-repeat analysis, the chemical similarity of each individual amino acid is low between individual FG-repeats, but the prediction of secondary structure features is well conserved (see Fig. S1B).

### Small-angle X-ray scattering analysis reveals an elongated, rod-like structure of F- and FG-repeats.

**FIG 6:**
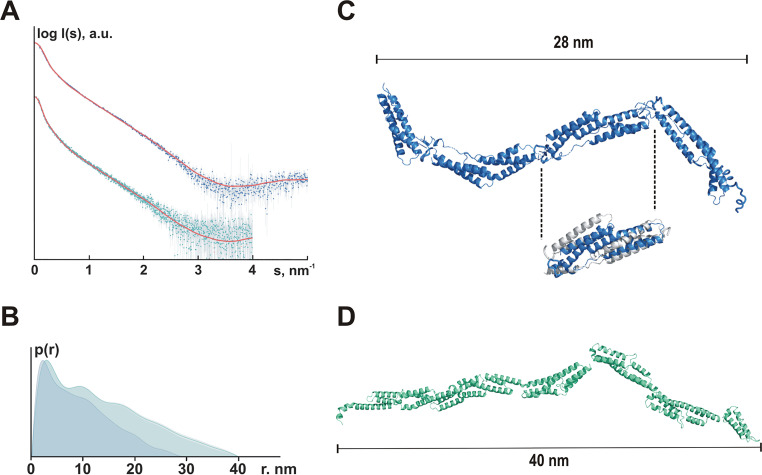
Organization of F- and FG-repeats in solution. (A) Experimental SAXS data from the Embp F-repeats construct (blue dots with error bars) and the Embp FG-repeat construct (cyan dots with error bars) fitted (red lines) with scattering computed from the rigid body models shown in Fig. S4A and B, respectively. For the F-repeats, the radius of gyration *R*_g_ estimated from Guinier approximation was 7.0 ± 0.2 nm, and the maximum dimension *D*_max_ was 28 nm; for the FG-repeats, the *R*_g_ was 10.7 ± 0.5 nm, and the *D*_max_ was 40 nm. (B) Pair distance distribution functions computed from the F-repeats (blue, *D*_max_ = 28 nm) and the FG-repeats (cyan, *D*_max_ = 40 nm) SAXS data. (C) Rigid body model of four F-repeats. Despite using several modeling approaches, it was not possible to obtain a satisfactory fit at higher angles (2 to 5 nm^−1^), although the overall shape resembled the respective *ab initio* model. Assuming that the structure of the individual F-repeats in solution was different from that in the crystal, the F-repeat structure was refined to fit the higher angle scattering data using the program SREFLEX (69). The RMSD between the original structure and the refined model (cutout: gray, F-repeat according to crystal structure analysis; blue, SREFLEX-refined F-repeat model) was 0.57 nm. RANCH (31) was then used to generate a model consisting of four repeats of the refined structure (best fit, χ^2^ = 2.24). Fits were computed by CRYSOL (68). (D) Rigid body model of six FG-repeats. RANCH (31) was used to generate a model consisting of six FG-repeats (best fit, χ^2^ = 1.07). Fits were computed by CRYSOL (68). For the rigid body model, no refinement of the 6GV5 model was required, suggesting that the crystal structure of the domain is preserved in solution.

Aiming at getting insights into the structural organization of repetitive F- and FG-repeats in solution, synchrotron small-angle X-ray scattering (SAXS) data were collected from Embp constructs corresponding to four F-repeats (632 aa residues, 70.4 kDa) and six FG-repeats (765 aa residues, 85.5 kDa) to characterize the shape of Embp in solution. Radius of gyration (*R*_g_; Fig. 6A) and pair distance distribution function [p(r); Fig. 6B] point to a rod-shaped organization of F- and FG-repeats. The pair distance distribution functions p(r) of both constructs revealed skewed profiles (typical for elongated particles) displaying sequential maxima corresponding to repeating domain structures (Fig. 6B). Indeed, the overall protein shapes reconstructed *ab initio* with the program GASBOR (30) yielded extended models consisting of multiple domains (see Fig. S4). Rigid body modeling was further utilized to represent F- and FG-repeat-containing constructs as interconnected assemblies of individual domains. For the F-repeats construct, the structure of the first F-repeat (PDB entry code 6GV8) was used as a building block, and four repeats were attached one after the other according to the construct’s sequence. Using RANCH (31) and CRYSOL fitting, the best model (χ^2^ = 2.24) displayed an extended shape with a *D*_max_ of 21.2 nm (Fig. 6C), well agreeing with the *ab initio* shape (see Fig. S4A).

10.1128/mBio.01612-20.4 FIG S4 (A) *Ab initio* model of four F-repeats using the GASBOR software package. Best fit, χ^2^ = 2.24. (B) *Ab initio* model of six FG-repeats using the GASBOR software package. Best fit, χ^2^ = 1.07. Download FIG S4, TIF file, 1.8 MB. Copyright © 2020 Büttner et al. 2020 Büttner et al. This content is distributed under the terms of the Creative Commons Attribution 4.0 International license.

A rigid model of six FG-repeats was established by using the crystal structure of one FG-repeat (PDB entry code 6GV5) as a building block, and putative models were generated as described above for the F-repeat. Similar to the F-repeat containing construct, the best model for FG-repeat containing protein (χ^2^ = 1.07, Fig. 6D) indicates that the six FG-repeats form a rather elongated structure with a *D*_max_ of 34.8 nm (Fig. S4B).

### rF- and rFG-repeats directly interact with fibronectin.

In order to test the hypothesis that predicted F- and FG-repeats could represent minimal functional Embp units, the recombinant rF- and rFG-repeat proteins, representing one F-repeat and one FG-repeat, were tested for Fn-binding properties. In a solid-phase, enzyme-linked immunosorbent assay (ELISA)-format binding assay, the rF- and rFG-repeats both showed binding to surface-immobilized Fn in a dose-dependent manner (see Fig. S5). This indicated that, despite significant structural differences, both repeat units possess Fn binding activity. To further characterize rF and rFG interactions with Fn, Fn-binding properties of both proteins were analyzed in surface plasmon resonance (SPR). Dose-dependent binding to surface-immobilized full-length Fn was measured (see Fig. S6A and B) validating results from ELISA-binding studies. Importantly, the *K_D_* for rF-repeat binding to Fn was determined to be approximately 4.0 × 10^−6^ M, which is substantially higher compared to the *K_D_* value obtained for the rFG-repeat, i.e., 1.6 × 10^−9^ M (Table 1).

**TABLE 1:** SPR-derived binding constants of Embp repeat-fibronectin interactions

Ligand	Analyte	Dissociation constant (*K_D_*) [M]
Fn	rF-repeat	4.0 × 10^−6^
Fn	rFG-repeat	1.6 × 10^−9^
rFN12-14	rF-repeat	2.9 × 10^−5^
rFN12-14	r2F-repeats	1 × 10^−5^
rFN12-14	r4F-repeats	2 × 10^−5^
rFN12-14	rFG-repeat	1.3 × 10^−7^
rFN12-14	r2FG-repeats	5 × 10^−7^
rFN12-14	r5FG-repeats	1 × 10^−5^

10.1128/mBio.01612-20.5 FIG S5 Binding of rF-repeat and rFG-repeat to immobilized fibronectin. Human fibronectin was used to coat the surfaces of 96-well microtiter plates. After blocking, wells were incubated with increasing amounts of the His_6_ fusion proteins rF-repeat or rFG-repeat. Bound recombinant proteins were detected by mouse anti-His_6_ IgG and alkaline phosphatase-conjugated anti-mouse IgG. Uncoated wells served as a negative control. Both Embp fragments bind in a dose-dependent manner to the conditioned surface. Download FIG S5, TIF file, 2.3 MB. Copyright © 2020 Büttner et al. 2020 Büttner et al. This content is distributed under the terms of the Creative Commons Attribution 4.0 International license.

10.1128/mBio.01612-20.6 FIG S6 Surface plasmon resonance analysis of rF- and rFG-repeat binding to fibronectin. Human fibronectin was immobilized on a C1 sensor chip (Biacore, Uppsala, Sweden) and binding of rF-repeat (7.8 to 62.6 μg/ml) and rFG-repeat (3.1 to 25 ng/ml) was analyzed. (A) Sensogram representing dose-dependent binding of rF-repeat to Fn. Based on the experiment, a binding constant *K_D_* of 4.0 × 10^−6^ M was calculated. (B) Sensogram representing dose-dependent binding of rFG-repeat to Fn. Based on the experiment, a binding constant *K_D_* of 1.6 × 10**^−^**^9^ M was calculated. Download FIG S6, TIF file, 0.5 MB. Copyright © 2020 Büttner et al. 2020 Büttner et al. This content is distributed under the terms of the Creative Commons Attribution 4.0 International license.

Experimental evidence supports the idea that Embp binds to the type III repeat 12 (FN12) of Fn (22). To confirm this hypothesis, binding of rF- or rFG-repeats to the recombinant ligand rFN12-14 was tested using SPR. The data revealed dose-dependent binding of both, rF and rFG to immobilized rFN12-14 (see Fig. S7A and B), unambiguously showing that both recombinant repeats interact with identical or very closely adjoining Fn-regions. Intriguingly, the rF-repeat again exhibited higher *K_D_* values (2.9 × 10^−5^ M) compared to the rFG-repeat (1.3 × 10^−7^ M) (Table 1).

10.1128/mBio.01612-20.7 FIG S7 Surface plasmon resonance analysis of rF- and rFG-repeat binding to rFN12-14. rFN12-14 was immobilized on a C1 sensor chip and binding of rF-repeat (15.6 to 125 ng/ml) and rFG-repeat (94 to 750 ng/ml) was analyzed. All experiments were performed in triplicates. (A) Sensogram representing dose-dependent binding of rF-repeat. Based on the experiment, a binding constant *K_D_* of 2.9 × 10^−5^ M was calculated. (B) Sensogram representing dose-dependent binding of rFG-repeat. Based on the experiment, a binding constant *K_D_* of 1.3 × 10**^−^**^7^ M was calculated. Download FIG S7, TIF file, 1.9 MB. Copyright © 2020 Büttner et al. 2020 Büttner et al. This content is distributed under the terms of the Creative Commons Attribution 4.0 International license.

Evaluation of the contribution of multiple F-repeats or FG-repeats to Embp-Fn interactions was performed by testing binding of recombinant proteins representing either two or four F-repeats or two or five FG-repeats to rFN12-14, respectively. Interestingly, multiple F- or FG-repeats also showed dose-dependent binding, although they did not exhibit higher binding affinities (i.e., lower *K_D_* values) compared to the single repeats (Table 1; see Fig. S8).

10.1128/mBio.01612-20.8 FIG S8 Surface plasmon resonance analysis of interactions between immobilized rFN12-14 and recombinant Embp fragments. (A and B) Binding of recombinant proteins consisting of two and four F-repeats, respectively. *K_D_* values for two repeats (1 × 10^−5^ M) and for four repeats (2 × 10^−5^ M) are similar to *K_D_* values estimated for a single F-repeat. (C and D) Binding of recombinant proteins consisting of two and four FG-repeats were used. Again, the calculated *K_D_* values for two repeats (5 × 10**^−^**^7^ M) and five FG-repeats (1 × 10**^−^**^5^ M) are similar to the *K_D_* values obtained for a single FG-repeat. Download FIG S8, TIF file, 0.7 MB. Copyright © 2020 Büttner et al. 2020 Büttner et al. This content is distributed under the terms of the Creative Commons Attribution 4.0 International license.

**FIG 7:**
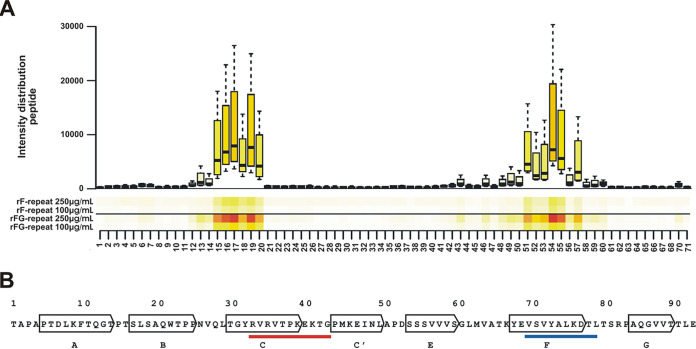
Identification of rF- and rFG-repeat binding sites within Fn type III repeat 12. (A) One-amino-acid overlapping 10-mers were immobilized on a microchip. The surface was then probed with fluorescence labeled rF- and rFG-repeats. Both recombinant Embp fragments demonstrated binding to almost the same peptides as shown in the heatmap. (B) Mapping of rF- and rFG-binding peptides onto the amino acid sequence of FN12. Arrows indicate seven β-sheets of FN12 (A to G) (32). The red underlined sequence indicates a projection of peptides with rF- and rFG-repeat binding activity located within the C β-sheet (aa 33 to 43). The blue underlined sequence indicates a projection of peptides with rF-repeat and rFG-repeat binding activity located within the F β-sheet (aa 70 to 79). The amino acid numbering refers to the FN12 sequence, as outlined in Sharma et al. (32).

**FIG 8:**
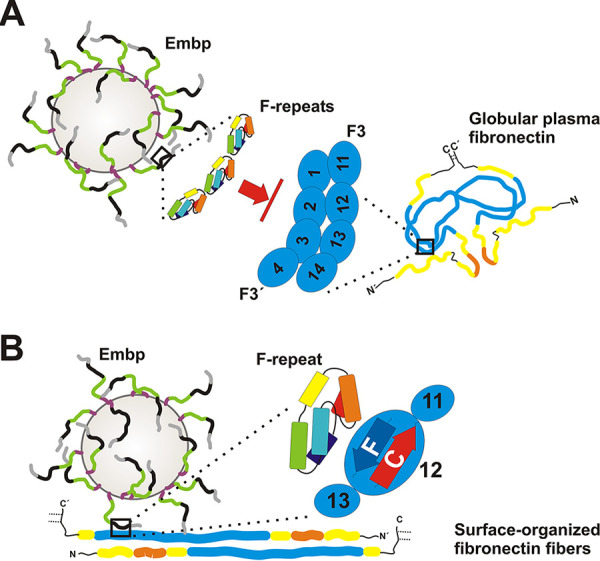
Graphical summary of Embp-mediated S. epidermidis interactions with fibronectin. S. epidermidis displays FG-repeat (green)- and F-repeat (black)-containing Embp on the cell surface of S. epidermidis (4), according to SAXS data most likely organized in elongated fibers. (A) Embp-mediated S. epidermidis interactions with globular Fn. The compact globular architecture of the soluble Fn-dimer is stabilized via intermolecular interactions, essentially involving FN2-3 of one Fn molecule (zoom-in right; F3′) and FN12 of the second molecule (zoom-in right, F3) (33). F-repeats (zoom-in left) or FG-repeats (not shown) possess Fn-binding activity through interactions with FN12. F-repeat and FG-repeat binding sites in FN12 are blocked by intramolecular Fn-Fn interactions, preventing Embp-mediated binding to globular Fn and its recruitment to the bacterial cell surface. (B) Embp-mediated S. epidermidis interactions with immobilized Fn. During surface deposition, Fn dimers become elongated by resolving intramolecular interactions and additional structural rearrangements of the F3 domain (73). As a consequence, F-repeat and FG-repeat binding sites within FN12 become accessible (i.e., within β-strands C and F; zoom-in), thus allowing S. epidermidis to adhere to Fn-conditioned surfaces. This process is fostered by additional, as-yet-uncharacterized S. epidermidis interactions with Fn. The figure is not drawn to scale.

To pinpoint the critical residues in FN12 that mediate binding to the F- and FG-repeats, a peptide mapping strategy was employed. To this end, a library of consecutive peptides spanning the entire FN12 repeat was synthesized. Each peptide contained 10 aa that overlapped by 1 aa. This peptide library was immobilized on a microarray and incubated with fluorescence-labeled rF- or rFG-repeats. These experiments identified several peptides within FN12 exhibiting both, rF- and rFG-repeat binding activity (Fig. 7A). From these, two minimal common sequences with rF- and rFG-binding activity were deduced, containing 15 and 16 aa, respectively. Mapping of these peptide positions onto the crystal structure of FN12-14 (32) showed that the 15-meric peptide (VQLTGYRVRVTPKEK) mapped to aa 25 to 40, located at the connecting loop between β-sheets B and C and within β-sheet C of FN12 (Fig. 7B). The second, 16-meric peptide (VATKYEVSVYALKDTL) projects to residues aa 64 to 80 of β-sheets F, G, and their connecting loop within FN12 (Fig. 7B). Both identified binding sites have thus far not been implicated in interactions with bacterial Fn-binding proteins. FN12 regions with Embp-binding activity have previously been demonstrated to be involved in intramolecular interaction between FN12 and FN2-3 and thereby in the stabilization of a folded globular structure of Fn in solution (Fig. 8). It is noteworthy that in in the folded state they are not accessible to other ligands (33).

## DISCUSSION

Interactions with host ECM components are a crucial step in colonization and infection establishment of many bacteria, and well over 100 bacterial cell surface proteins with Fn-binding activity have been identified so far (34). Among those, a predominant interaction mechanism is binding to the N-terminal Fn type I domain (F1). E.g., two well-studied proteins, S. aureus FnBPA and S. dysgalactiae FnBB, bind to ^2-5^F1 and ^1-2^F1 repeats, respectively, and both proteins employ a similar tandem β-zipper mechanism (25, 35). By using a Fn-binding mechanism involving FN12, Embp thus clearly represents one of the rare examples in which a bacterial adhesin binds to C-terminal Fn type III domain (Fig. 8). To our knowledge, this has only been demonstrated for B. henselae surface protein Pap31 (36), S. enterica adhesin ShdA (37) and the pneumococcal fibronectin-binding proteins PavA and PavB (38). In extension to previous work (22), evidence supports the idea that two minimal structural repeat units F and FG of Embp mediate binding to full-length Fn, most probably via interactions with two peptides located within FN12, and unrelated to the Hep-II binding site in FN13. Mechanistically, Embp is hereby different from FN13 binding proteins Pap31 and ShdA, which bind specifically to the ABE face of the triple-stranded β-sheet (39) of FN13 (37). Thus, Embp binding to Fn represents a novel principle in pathogen-ECM interactions. Noteworthy, deletion of type III subdomains FN11 to FN14 resulted in a drastically reduced adherence of Embp-expressing S. epidermidis to conditioned surfaces compared to the full-length Fn, and this finding is in line with a previously published report attributing a crucial role to the C-terminal part of Fn for S. epidermidis adherence to Fn (26). The residual adherence activity, however, point toward additional, as-yet-uncharacterized modes of S. epidermidis-Fn interactions. These may include interactions between Embp and FN12 to 14-independent Fn regions (e.g., FN7 to 10) or independent S. epidermidis cell surface structures possessing Fn-binding activity (e.g., wall teichoic acids [WTA]) (20). Of note, the very C-terminal F1 subdomains might also be relevant for S. epidermidis-Fn interactions. Possible additional modes of Embp-Fn interactions, and the specific contribution of F- and FG-repeats here needs to be explored in future studies.

Fn exists in multiple conformations and organizational states, dependent on its molecular composition, localization in the body tissues and the types of cells it is associated with. The two major Fn forms are a compact globular and an extended fibrillous conformation (Fig. 8). Well-characterized S. aureus FnBPA, as well as additional bacterial Fn binding proteins, facilitates interactions with Fn irrespective of the conformational state through binding to N-terminal Fn type I repeats. In fact, S. aureus particularly depends on binding to globular Fn via FnBPA to enter into endothelial cells (40), a process that essentially contributes to infective endocarditis pathogenesis (41). In sharp contrast, several aspects suggest that immobilization of Fn structure and resulting conformational changes (42, 43) have a profound impact on interactions with Embp and Embp-mediated S. epidermidis adherence to Fn. Interactions of recombinant Embp fragments and Embp-producing S. epidermidis became evident with surface-deposited Fn, whereas soluble rFN12-14 failed to bind to immobilized Embp fragments. Moreover, Embp-producing S. epidermidis was unable to recruit soluble Fn, while it readily bound to surface-immobilized Fn. It is well accepted that the formation of Fn-fibrils results in a number of conformational changes within the molecule (44, 45) and, during this process, stabilizing intramolecular interactions between FN12 and FN2-3 are lost (Fig. 8) (33, 46). In addition, the extension of the Fn molecule during fibrillogenesis leads to the exposure of cryptic epitopes within the type III repeats (47). Both aspects, together with the biochemical evidence for an Embp binding site in FN12 reported here, can be regarded as a possible molecular explanation for the differential binding of Embp to Fn depending on the Fn organizational state (Fig. 8). Future studies will need to characterize the impact of the Fn surface organization on Embp interactions in more detail, e.g., by using surfaces allowing to specifically direct Fn organization (i.e., globular versus elongated) (48). Analysis of such dynamic structure-function relationships in Embp-Fn interactions will be of key interest in the future, since they might potentially serve S. epidermidis as a means to target and colonize dedicated host niches during commensalism (e.g., the human nasal cavity) or invasion (e.g., ECM-covered implants). Moreover, given that FN12-14 is important for Fn fibrillogenesis (49), the impact of Embp-Fn interactions on Fn fibril formation needs to be further studied.

Thus far, only limited knowledge about the structural organization of Embp is available, essentially being derived from high-resolution crystal structures of two repeats R7-R8 from S. aureus protein EbhA, a homologue of Embp (29). Combined with data from small-angle X-ray scattering (SAXS) experiments, repeating units were supposed to represent core structural elements of EbhA, and it was estimated that the protein forms a rod-like overall structure of 320 nm total length. Although the overall amino acid sequence identity with Embp is only 30%, the structural data for the FG-repeat at 1.55-Å resolution reveals high structural homology to EbhA and strict conservation of all major structural key elements. Here, by strong interactions between subsequent FG-repeats, the dipole character of the FG-repeats likely supports the SAXS-deduced, elongated rod-like structure of repetitive FG-repeats.

Compared to the FG-repeat, the crystal structure of the F-repeat reveals some similarities but also significant differences. Most importantly, the F-repeat misses a common long central helix, being relevant for structural integrity of the FG-repeat. Within the F-repeat, however, cohesion between α-helical bundles is supported by a so-called Sandwich-loop, a structural feature not described before. Based on the high-resolution structure, it appears that the F-repeat part of Embp is likely to be less tight and rigid than the FG-repeat containing region. This assumption matches our results from SAXS-analysis, providing evidence for a more flexible, but still elongated overall architecture.

Importantly, although similar to F1 repeats, F3 repeats, including FN12, also consist of multiple antiparallel β strands, the overall α-helical organization of rF- and rFG-repeats suggests that the general mode of interactions with FN12 is mechanistically unrelated to that of the tandem β-zipper mechanism described for S. aureus FnBPA (25). Future work will therefore address in detail the structural basis of F- and FG-repeat complex formation with FN12. Such work could also shed light on the molecular basis for the differences in binding affinities observed for F- and FG-repeats and clarify which extended multivalent binding modes are involved in Embp-Fn interactions.

Taken together, the findings presented in this study significantly extend our mechanistic understanding of giant 1-MDa Embp, S. epidermidis surface colonization, and subsequent establishment of persistent infections. Previous studies have shown that Embp mediated biofilm formation is of importance during host-pathogen interactions by protecting S. epidermidis from clearance by macrophages (4, 22, 50). The novel mechanism underlying Embp binding to Fn may support additional modes of host-pathogen interactions, e.g., through binding to FN12 Embp has the potential to interfere with Fn fibrillogenesis (49) and matrix assembly.

## MATERIALS AND METHODS

### Bacterial strains, culture conditions, and general procedures.

Unless indicated, staphylococci were grown in tryptic soy broth (TSB; Becton Dickinson, Sparks, MD). Escherichia coli strains were grown in lysogeny broth (LB; Becton Dickinson) at 37°C. Bacterial strains were stored at –80°C in cryobank tubes (Mast, Reinfeld, Germany) (Table 2).

**TABLE 2:** Cell lines, strains, and bacteriophages used in this study

Cell line or bacterial strain	Properties/genetics	Source or reference
Cell line		
HEK293	Adherent embryonic kidney cell line for transfection and isolation of Fn and Fn isoforms.	74
		
Bacterial strains		
E. coli TOP10	Cloning host; genotype F^–^ *mcrA* Δ(*mrr-hsdRMS-mcrBC*) ϕ80*lacZ*ΔM15 Δ*lacX74 recA1 araD139* Δ(*araleu*)*7697 galU galK rpsL* (Str^r^) *endA1 nupG*	Invitrogen, Carlsbad, CA
E. coli BL21 AI	Host for recombinant protein expression; genotype F^–^ *ompT hsdS*_B_(r_B_^–^ m_B_^–^) *gal dcm araB*::T7RNAP-*tetA*	Invitrogen, Carlsbad, CA
E. coli BL21 STAR	Host for recombinant protein expression; genotype F^–^ *ompT hsdS*_B_ (r_B_^–^ m_B_^–^) *galdcmrne131* (DE3)	Invitrogen, Carlsbad, CA
T7 Express Crystal E. coli	Methionine auxotrophic host for recombinant expression of the selenomethionine-derivatized rF-repeat; genotype: *fhuA2 lacZ*::*T7 gene1* [*lon*] *ompT gal sulA11R*(*mcr-73*::miniTn*10−*TetS)*2* [*dcm*] *R*(*zgb-210*::Tn*10−*TetS) *endA1 metB1 Δ*(*mcrC-mrr*)*114*::*IS10*	New England Biolabs, Ipswich, MA
S. epidermidis 1585	Clinical S. epidermidis wild-type isolate from a port catheter infection; *icaADBC* and *aap* negative; biofilm negative	22, 75
S. epidermidis 1457-M12	Tn*917* insertion in *purR*; S. epidermidis recipient for plasmid electroporation	76
S. epidermidis 1585Δ*embp*	Markerless *embp* knockout mutant derived from S. epidermidis 1585	This study
S. epidermidis 1585*P*_xyl/tet_*embp*	Derivative of S. epidermidis in which *embp* is placed under the control of an anhydrotetracycline-inducible element	22
S. aureus RN4220	Restriction-deficient mutant derived from S. aureus RN450	77
*S. carnosus* TM300	Surrogate host	78
ΦA6C	Transduction from/into S. epidermidis strains	53

### Generation of an *embp* deletion mutant in *S. epidermidis* 1585.

Nucleotide positions refer to the S. epidermidis 1585 *embp* coding sequence. Temperature-sensitive shuttle vector pKO_R1 (51) was used to generate a markerless *embp* knockout in S. epidermidis 1585. To this end, a 959-bp fragment starting 1,013 bp upstream of the *embp* start codon (primers 1 and 2) and another 846-bp fragment located within the *embp* coding sequence (nucleotides [nt] 29639 to 30483) (primers 3 and 4) were amplified from chromosomal S. epidermidis 1585 DNA. PCR products were purified by gel extraction, digested using PstI (New England Biolabs, Ipswich, MA), and ligated using T4 DNA ligase (New England Biolabs). The ligation reaction product was used as a template to amplify fused 5′- and 3′ fragments (primers 1 and 4). Amplicons were then introduced into pKO_R1 by BP clonase (Invitrogen, Carlsbad, CA). Cloning product was analyzed by restriction digest, PCR and sequencing. Plasmid pKO_*embp* was then transferred into restriction-deficient S. aureus RN4220 via electroporation (52). Electroporation of the extracted plasmid into S. epidermidis 1457-M12 finally enabled phage transduction (53) into target strain S. epidermidis 1585.

Allelic replacement was carried out according to the procedure described elsewhere (51). The successful knockout was identified using PCR strategy employing appropriate oligonucleotides (primers 5 and 6). Inactivation of *embp* was functionally tested using dot blot immune analysis. To this end, cell surface associated proteins were prepared from S. epidermidis 1585 and 1585Δ*embp* grown in the presence of tigecycline for induction of Embp production (50). Detection of Embp was carried out by using Embp-detecting antiserum (22) (see Fig. S9).

10.1128/mBio.01612-20.9 FIG S9 Construction and validation of S. epidermidis *embp* knock-out mutant 1585Δ*embp*. (A) Schematic figure showing the strategy to knock out *embp* in S. epidermidis 1585 by an allele replacement strategy. Numbers indicate the relative nucleotide positions according to the *embp* reading frame in S. epidermidis 1585. Oligonucleotides 5 and 6 were used to verify the correct genetics in the mutant. (B) PCR analysis of wild-type S. epidermidis 1585 and corresponding mutant 1585Δ*embp*. Chromosomal DNA was used as a template in a PCR reaction in which primers binding up-stream and down-stream of *embp* (oligonucleotides 5 and 6, respectively) were added. Due to the size of the *embp* CDS, no amplicon was retrieved with DNA from wild-type S. epidermidis 1585. Sequencing of the amplicon obtained using DNA from 1585Δ*embp* demonstrated correctness of the knock-out mutant. (C) Phenotypic confirmation of the *embp* deletion. Dot immunoassay demonstrating the loss of Embp production in S. epidermidis 1585Δ*embp*. Bacteria were grown in the presence of 125 ng/ml tigecycline (i.e., *embp*-inducing conditions). After 6 h of growth, the bacteria were harvested, and the cell surface-associated proteins were extracted by incubating the bacteria in 1× LDS buffer (Invitrogen) at 70°C for 10 min. Then, 10 μl of culture supernatant and the cell pellet were loaded onto a polyvinylidene difluoride membrane. Embp was detected using an Embp-specific rabbit antiserum (anti-rEmbp6599), and a peroxidase-coupled anti-rabbit IgG. S. epidermidis 1585 served as positive control. Download FIG S9, TIF file, 0.5 MB. Copyright © 2020 Büttner et al. 2020 Büttner et al. This content is distributed under the terms of the Creative Commons Attribution 4.0 International license.

### *In trans* expression of Embp fragments in *S. epidermidis*.

Two DNA fragments encoding five F-repeats (nt 7905 to 11079) and nine FG-repeats (nt 13516 to 17079) were amplified from S. epidermidis 1585 chromosomal DNA and fused to the natural YSIRK-motif containing export signal (nt 1 to 365 of *embp*) and the 3′ end of *embp* (encoding DUF1542 domains and a putative transmembrane region [nt 28239 to 30609]). Recombinant DNA fragments were ligated into pCR4 (Invitrogen) containing a modified multiple cloning site (referred to as pCR4-mod; Table 3). The correctness of cloning was tested by restriction enzyme analysis, PCR, and sequencing. For expression in staphylococci, recombinant *embp* fragments were amplified (primers 7 and 8) and subcloned into pHB2_C_. pHB2_C_ is a derivative of pCN57 (54) in which the *ermB* cassette was replaced by *cat194*, and an anhydrotetracycline-inducible promoter (55) was inserted. The resulting plasmids are referred as pEmbp_5F and pEmbp_9FG. Both plasmids were transformed into S. aureus RN4220 and transferred to S. epidermidis and S. carnosus. The correct transformation of the target strains was verified by PCR and restriction analysis of purified plasmids. The functionality of the expression plasmids was confirmed by detection of Embp fragments using immunoblotting and immunofluorescence assay.

**TABLE 3:** Plasmids used in this study

Plasmid	Features	Source or reference
pET302/NT-His_TEV-rF-repeat	Recombinant Embp fragment corresponding to aa 2569 to 2738	This study
pDEST17-rFG-repeat	Recombinant Embp fragment corresponding to aa 6777 to 6902	This study
pDEST17-2xrF-repeat	Recombinant Embp fragment corresponding to aa 2569 to 2908	This study
pDEST17-2xrFG-repeat	Recombinant Embp fragment corresponding to aa 6777 to 7028	This study
pDEST17-4xrF-repeat	Recombinant Embp fragment corresponding to aa 2569 to 3248	This study
pDEST17-5xrFG-repeat	Recombinant Embp fragment corresponding to aa 6777 to 7406	This study
pKOR1	Temperature-sensitive shuttle vector for allele replacement; Cm^r^	51
pKO_embp	Generation of markerless deletion of *embp*	This study
pCR4-mod	pCR4 carrying a modified multiple cloning site (5′-CACCATACCCGGGATAAACGCTAGCATCGGTACGCGTAATGCACTCGAGATAAATGGTACCAAC-3′)	This study
pCN57	Shuttle vector carrying *gfp*; Erm^r^	54
pCN50	Used as a template to amplify *cat194*	54
pALC2073	Used as a template to amplify P*_xyl/TetO_*	55
pHB2_C_	Derivative of pCN57; carries *cat194* and P*_xyl/TetO_*	This study
pEmbp_5F	*In trans* expression of aa 2636 to 3693	This study
pEmbp_9FG	*In trans* expression of aa 4506 to 5693	This study
FN-YPet/pHLSec2	Wild-type Fn cDNA corresponding to RefSeq NP_997647 and harboring additional mYFP inserted between FN6 and FN7	28
FNΔ^III^11-14/pHLSec2	Fn cDNA corresponding to RefSeq NP_997647 and carrying additional mYFP following FN10 and a deletion of FN11 to FN14	28

### Heterologous expression of recombinant proteins.

The sequences encoding single F- and FG-repeats were amplified from chromosomal DNA of S. epidermidis 1585 (primers 9 and 10 [F-repeat] and primers 13 and 14 [FG-repeat]), respectively. The PCR product for the F-repeat was used for Gibson Assembly (New England Biolabs), inserting it into expression vector pET302/NT-His (Invitrogen). The FG-repeat fragment was cloned into pENTR/d-TOPO (Invitrogen), and then Gateway technology (Invitrogen) was used to introduce the amplicon into the expression vector pDEST17 (Invitrogen). All cloning steps were performed in E. coli DH5α (Invitrogen). Finally, correct insertion of the *embp* sequences were verified using restriction analysis and sequencing. Cloning of multiple F- or FG-repeats was carried out as described for the F-repeat using the oligonucleotides listed in Table 4.

**TABLE 4:** Oligonucleotides used in this study

No.	Oligonucleotide	Sequence (5′–3′)
1	for_attP	GGGGACAAGTTTGTACAAAAAAGCAGGCTTAACATTTTTATGCAACAAG
2	5rev_Pst	GCTACATCTGCAGCAATTTATTTGTTCTAAACAATAATATCAC
3	3for_Pst	ATGTAGCCTGCAGTTAGAAAAAGTCGAGCATGCTC
4	3rev_attP	GGGGACCACTTTGTACAAGAAAGCTGGGTAGGATTGAATGAATATCCT
5	embp_−255_for	CCGAAGTGCTTGTGCG
6	embp_+510_rev	CCGAAGTGCTTGTGCG
7	inf_Eco_Embp_for	GGTACCGAGCTCGAATTCAAATGCTATTGTGATAAATGAAGAG
8	inf_Asc_Embp_rev	TGCATTTAGAATAGGCGCGCCATATATTTTACTTTTTAGAAC
9	1F_fwd	TGCATCATCATCATCATCACGTGGAAAACCTGTATTTTCAGGGCACTAAAGTTAATAAAACCGAATTAATC
10	1F_rev	AATATCATCGATCTCGAGCGTTAGTTGCTTTTAGCTTCAAC
11	2F_rev	TTAATTACTTTTAGCATTAGTTAAAGCTTG
12	4F_rev	AATATCATCGATCTCGAGCGTTAATGTTGTTTTGCTTCG
13	1FG_for	CACCGGAGATCAAAAACTTCAAGATGC
14	1FG_rev	TTAATGAAGATTTTGTTCAGCAT
15	2FG_rev	TTACAATGAATCTTTTGCTTGAATGA
16	5FG_for	CACCGGAGAATCCAGATTTAAACA
17	5FG_rev	TTAATGTAAACTTTCTCTAGCATTTTGC

Single rF-repeat was expressed in E. coli BL21 STAR (Invitrogen). Expression of all of the constructs based on pDEST17 was carried out using E. coli BL21 AI (Invitrogen). Overnight cultures were diluted into fresh LB and grown at 37°C under shaking (200 rpm) conditions. The expression of rF- and rFG-repeat was induced by adding 1 mM isopropyl β-d-1-thiogalactopyranoside or arabinose (0.02% [wt/vol]) when an optical density (600 nm) of 0.6 was reached. Bacteria were grown for three more hours under inducing conditions. Finally, bacterial cells were harvested by centrifugation (6,000 rpm for 10 min at 4°C). The expression of selenomethionine-derivatized rF-repeat was performed as described in the manufacturer’s protocol for methionine auxotrophic T7 Express Crystal competent E. coli (New England Biolabs). The expression of recombinant fibronectin subdomains in E. coli was conducted as described elsewhere (27).

A transient mammalian cell expression system was used to generate fibronectin derivates. Briefly, adherent HEK293 cells were transfected with mammalian cell expression plasmids using a polyethylenimine (PEI) method (25,000 MW; Polysciences, Warrington, PA) as described elsewhere (56). These plasmids code for Fn derivates either harboring the full-length Fn or a truncated version lacking FN11 to 14. Both constructs include a YFP (28). Supernatants were collected 5 days after transfection of the HEK293 cells, centrifuged, and filtered.

### Purification of recombinant proteins.

Bacteria from expression cultures were suspended in standard binding buffer for Ni-affinity chromatography and lysed by sonification. After clearing of the lysate, the proteins were purified by Ni-affinity chromatography using an ÄKTA purifier 10 system (GE Healthcare, Uppsala, Sweden). Cleavage of the N-terminal His tag was done by using TEV protease (Sigma-Aldrich, St. Louis, MO) according to the manufacturer’s recommendations. Additional purification steps via cation- or anion-exchange chromatography or size exclusion chromatography were performed if necessary. The purity of the recombinant expressed proteins was tested by Coomassie blue-stained SDS-PAGE.

HEK293-derived Fn derivates were purified from the supernatants by gelatin agarose affinity chromatography method as described elsewhere (28). The success of the purification was proven by dot immunoassay using monoclonal anti-GFP antibody (Abcam, Cambridge, MA).

### Crystallization, data collection, and structure determination.

The FG-repeat crystals were grown by sitting drop vapor diffusion technique by equilibrating a 1-μl drop containing equal volumes of 32 mg/ml protein and precipitant (condition E8, Morpheus HT-96 Screen; Molecular Dimensions) against a well containing 45 μl of precipitant. Crystals grew within 3 to 6 days at 294 K. Both the native F-repeat and selenomethionine-labeled F-repeat crystals were grown by the sitting-drop vapor diffusion technique by equilibrating a 1-μl drop containing equal volumes of 40 mg/ml protein and precipitant (condition E11, Morpheus HT-96 Screen; Molecular Dimensions) against a well containing 45 μl of precipitant. The setups were made by the Cartesian Honeybee916 system for protein crystallization (Genomic Solutions). Crystals grew within 5 to 7 days at 294 K.

All crystals were taken from the drop and flash cooled directly in a liquid N_2_ stream prior to data collection. X-ray diffraction data were collected at 100 K at the Petra III synchrotron storage ring (DESY, Hamburg, Germany). Native and Se-SAD data were collected at beamline P11, equipped with a Pilatus 6 M detector. The diffraction data were processed by using the XDS program package (57).

For the FG-repeat, the phase problem was solved by molecular replacement using a solvent-free version a partial structure (residues 73 to 198) of the cell wall-associated adhesion protein EbhA-R7-R8 (29) from Staphylococcus aureus (PDB code 2DGJ) as a starting model in phenix.phaser (58). The structure was improved and refined using phenix.refine (59) at 1.55 Å. For the F-repeat, the molecular replacement approach failed. No useful starting model was identified. Experimental phases were calculated by using a SAD approach with diffraction data from the Se-Met-variant of the F-repeat using the program package phenix.autosol (60), an experimental phasing pipeline that combines the programs HySS (Hybrid Substructure Search) for finding heavy-atom sites, Phaser or SOLVE for calculating experimental phases, and RESOLVE for density modification and model-building. The structure was improved and refined using the high-resolution diffraction data of the native F-repeat at 1.39 Å. The refinement was carried out for both the F- and the FG-repeat using phenix.refine (59) by iterative cycles of restrained maximum-likelihood refinement, and manual model rebuilding using COOT (61), Polygon (62), and MolProbity (63) were used for the validation of the final model. Data collection and structure refinement statistics are listed in Table S1A in the supplemental material. Figure 5 was generated using PyMOL (The PyMOL Molecular Graphics System, version 2.0; Schrödinger, LLC.).

10.1128/mBio.01612-20.10 TABLE S1 (A) Data collection, phasing, and refinement statistics. (B) SAXS data collection, analysis, and modeling. Download Table S1, DOCX file, 0.02 MB. Copyright © 2020 Büttner et al. 2020 Büttner et al. This content is distributed under the terms of the Creative Commons Attribution 4.0 International license.

### Small-angle X-ray scattering analysis.

The synchrotron radiation X-ray scattering data from four 1.0- to 4.1-mg/ml solutions of Embp F-repeats were collected at the EMBL beamline X33 on the DORIS III storage ring (DESY) (64). Using a Pilatus 1M-W detector at a sample-to-detector distance of 2.7 m and a wavelength (λ) of 0.15 nm, a range of momentum transfer 0.09 < s < 6.0 nm^−1^ was covered (s = 4π sinθ/λ, where 2θ is the scattering angle). To monitor for the radiation damage, 8 successive 15-s exposures of protein solutions were compared, and no damage was observed.

The synchrotron radiation X-ray scattering data from six 1.0- to 11.0-mg/ml solutions of Embp FG-repeats were collected at the EMBL beamline P12 on a PETRA III storage ring (DESY) (65). Using a Pilatus 2M detector at a sample-to-detector distance of 3.0 m and a wavelength (λ) of 0.124 nm, the range of momentum transfer 0.08 < s < 4.5 nm^−1^ was covered. The protein solutions were measured using a continuous flow cell capillary. To monitor for the radiation damage, 20 successive 0.05-s exposures of protein solutions were compared, and frames with statistically significant changes were discarded.

The data were normalized to the intensity of the transmitted beam and radially averaged; the scattering of the provided buffer was subtracted, and the difference curves were scaled for the protein concentration. No concentration-dependent interparticle interference effects were observed, the highest concentration data were used. The radius of gyration *R*_g_, the forward scattering I(0), the pair-distance distribution function of the particle p(r), and the maximum dimension *D*_max_ were derived using the automated SAXS data analysis pipeline SASFLOW (66). The molecular weights (MW) of Embp F- repeats were evaluated by comparison of the forward scattering to that from a bovine serum albumin (MW = 66 kDa) reference solution. The MW of the Embp FG-repeats was evaluated using a consensus Bayesian assessment approach (67).

*Ab initio* shape models were generated using the dummy residues modeling program GASBOR (30). This program represents the protein shape by a chain-like ensemble of dummy residues and employs simulated annealing to construct an interconnected model fitting the experimental data. For each data set, the *ab initio* modeling was done 10 times, and the best-fitting models were chosen.

Rigid body models were built using the program RANCH (31), which generated a pool of 10,000 randomized models based on the protein sequence and the repeat structures deposited to PDB ID numbers 6GV8 (F-repeat) and 6GV5 (FG-repeat). The scattering from these models was calculated with the program CRYSOL (68); given the atomic coordinates, the program minimizes the discrepancy in the fit to the experimental intensity by adjusting the excluded volume of the particle and the contrast of the hydration layer. The models with the lowest χ^2^ fits were chosen. The F-repeat structure was refined using the program SREFLEX (69), which uses normal mode analysis to estimate the flexibility of the rigid domains and improve the model agreement with experimental data at higher angles (2 to 5 nm^−1^).

The experimental SAXS data and the obtained models were deposited in the Small Angle Scattering Biological Data Bank (SASBDB [70]) under the the accession codes SASDJ92 (F-repeats) and SASDJA2 (FG-repeats). The data collection and analysis details are summarized in Table S1B.

### Analysis of Embp binding to fibronectin by ELISA, surface plasmon resonance, and peptide mapping.

Embp interaction with Fn was tested in an ELISA format assay. 96-well microtiter plates (Greiner, Frickenhausen, Germany) were incubated with 100 μl of Fn solution (100 μg/ml; Sigma-Aldrich) overnight. Contamination of human Fn by fibrinogen was ruled out by immunoblotting with anti-fibrinogen antibody (Sigma-Aldrich). Unbound Fn was removed by washing with phosphate-buffered saline including 0.05% Tween 20 (PBS/T; Merck, Darmstadt, Germany), and wells were blocked with protein-free blocking buffer (Pierce, Rockford, IL). Subsequently, increasing concentrations of the rF- or rFG-repeat were added to Fn-coated wells, followed by incubation at room temperature for 1 h. Plates were washed with PBS/T to remove unbound Embp fragments. Detection of Embp was done by using an Infinite 200 plate reader (Tecan, Männedorf, Switzerland). All binding assays were performed in three technical and at least two independent biological replicates.

Surface plasmon resonance (SPR) analysis of Embp-Fn interactions was carried out on a Biacore T200 instrument (BIACORE, Uppsala, Sweden). C1 sensor chip (BIACORE) was run at 25°C using HBS-EP running buffer (10 mM HEPES [pH 7.4], 150 mM NaCl, 3 mM EDTA, 0.005% Surfactant P20). Fibronectin (50 μg/ml; Sigma-Aldrich) was suspended in 10 mM sodium acetate at pH 5.0 and immobilized (∼1,000 response units [RU]) using Amine Coupling kit (BIACORE). To confirm binding of rF- and rFG-repeats (diluted in HBS-EP) to Fn, these proteins were injected at 40-μl/min flow rate (300-s injection plus+ 300-s dissociation). Surfaces were regenerated by applying a single pulse of NaOH (BIACORE).

Recombinant rFN12-14 (100 μg/ml) in 10 mM NaPO_4_ (pH 7.8) was immobilized (∼870 RU) on C1 sensor chip surfaces. To confirm binding of the rF- and rFG-repeat or multiple repeats (diluted in HBS-EP) to immobilized rFN12-14 those proteins were injected at a 40-μl/min flow rate (300-s injection plus 300-s dissociation). Double referencing (71) of all binding data was performed prior to further analysis.

To identify Embp-binding regions within the Fn type III repeat 12, a commercially available PepStar microarray (JPT, Berlin, Germany) was used. A total of 83 1-aa overlapping 10-meric peptides representing the FN12 primary amino acid sequence were synthesized. Triplicates of each of the peptides were spotted via a linker onto glass targets. The microarray was then probed with DyLight 650-labeled rF- and rFG-repeats at a range of concentrations from 1 to 250 μg/ml. Binding data were acquired by quantifying the fluorescence using GenePix (Molecular Devices, Sunnyvale, CA). For further data evaluation, the MMC2 values were determined. The MMC2 equals the mean value of each of three replicates on the microarray. Except for the coefficient of variation (CV), i.e., the standard deviation divided by the mean value is larger than 0.5; in this case, the mean of the two closest values (MC2) was assigned to MMC2. These values were than used to generate a signal to peptide plot.

### Recruitment of soluble fibronectin to the staphylococcal cell surface.

Overnight cultures of *S. epidermidis* 1585*P*_xyl/tet_*embp*, 1585Δ*embp*, and 1585Δ*embp* × pFNBA4 were grown overnight in TSB (Becton Dickinson) containing appropriate antibiotics at 37°C and with 200-rpm shaking. After 18 h, the cultures were diluted 1:100 in fresh media, and *embp* expression was induced by the addition of anhydrotetracycline (100 ng/ml; Sigma-Aldrich) to each culture. Bacteria were grown for 6 h under inducing conditions at 37°C with continuous shaking (200 rpm) and then harvested by centrifugation. Pellets were washed once in PBS and finally suspended in 1 ml of PBS. The cell suspensions were adjusted to identical optical densities, and 100-μl portions of the bacterial suspension were incubated with 100 μl of human Fn (10 μg/ml; Sigma-Aldrich) or bovine serum albumin (10 μg/ml; Serva, Heidelberg, Germany) for 1 h. Afterward, the bacteria were washed twice in PBS. Then, 10-μl portions of these bacterial suspensions were spotted onto glass coverslips and fixed using 20 μl of 1% paraformaldehyde solution (Merck). After blocking, bound Fn was stained with rabbit anti-Fn antibody (1:300 in PBS; Sigma-Aldrich) and Alexa 488-labeled anti-rabbit IgG (1:500 in PBS; Sigma-Aldrich). Coverslips were evaluated using confocal laser scanning microscopy with a TCS SP8 instrument (Leica, Wetzlar, Germany). All stacks were taken with a 0.2-μm distance, while the total thickness of each stack was no more than 20 μm.

### Analysis of bacterial adherence to surface-immobilized fibronectin and fibronectin subdomains.

Bacterial adherence assays (S. epidermidis 1585 and *S. carnosus* TM300) to Fn were performed as described elsewhere (22, 53). To determine bacterial adherence to surface-immobilized recombinantly expressed Fn subdomains, Immobilizer Nickel-Chelate microtiter plates (Nunc, Roskilde, Denmark) were used. Surface saturation was achieved at concentrations of 20 μg/ml. An uncoated surface was used as a negative control. Fn derivates purified from HEK293 cell culture supernatants were immobilized on 96-well microtiter plates (Greiner, Frickenhausen, Germany) overnight at 4°C.

Bacteria were grown for 6 h in TSB (supplemented with appropriate antibiotics), harvested by centrifugation, and washed in PBS. Bacteria were incubated for 1 h at room temperature on conditioned microtiter plates. After the removal of unbound bacteria, adherent cells were measured by using rabbit anti-S. epidermidis antibody (72) and anti-rabbit IgG-antibody-alkaline phosphatase conjugate (Sigma-Aldrich). After 30 min of incubation at 37°C with phosphatase substrate (1 mg/ml; Sigma-Aldrich), the enzymatic reaction was quantified by using an Infinite 200 plate reader (Tecan). All binding assays were performed using three technical replicates and at least two independent biological replicates.
