# Supplementary figures and images for: A Giant Extracellular Matrix Binding Protein of Staphylococcus epidermidis Binds Surface-Immobilized Fibronectin via a Novel Mechanism
Source: mBio. 2020 Oct 20;11(5):e01612-20. doi: 10.1128/mBio.01612-20 (PMC7587433; doi:10.1128/mBio.01612-20)

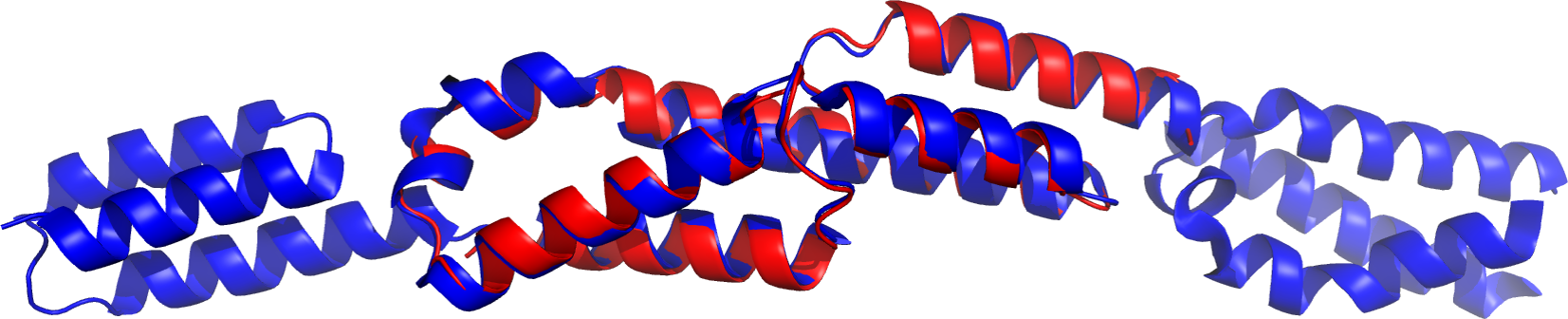

Supplement: FIG S3 [file mBio.01612-20-sf003.tif]

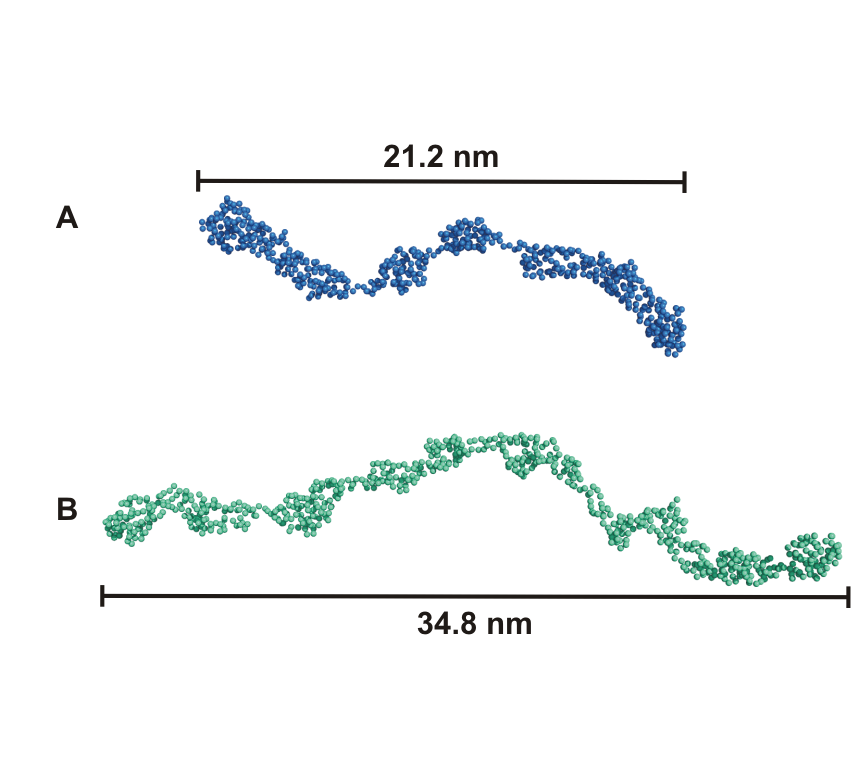

Supplement: FIG S4 [file mBio.01612-20-sf004.tif]

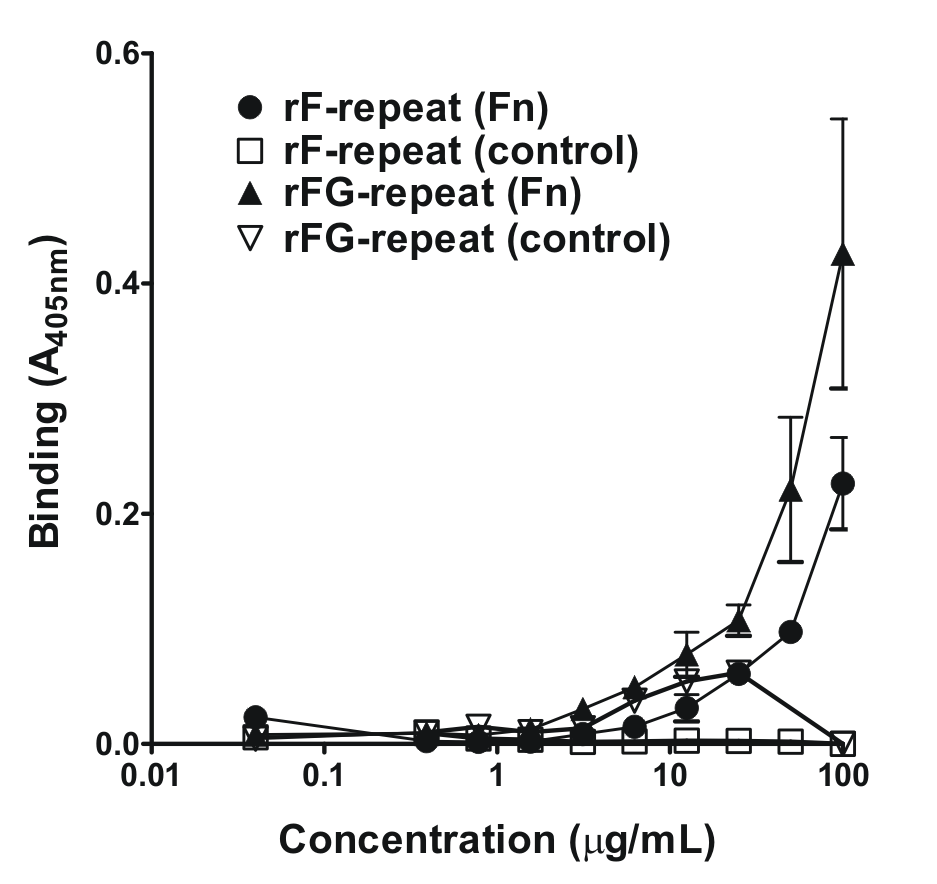

Supplement: FIG S5 [file mBio.01612-20-sf005.tif]

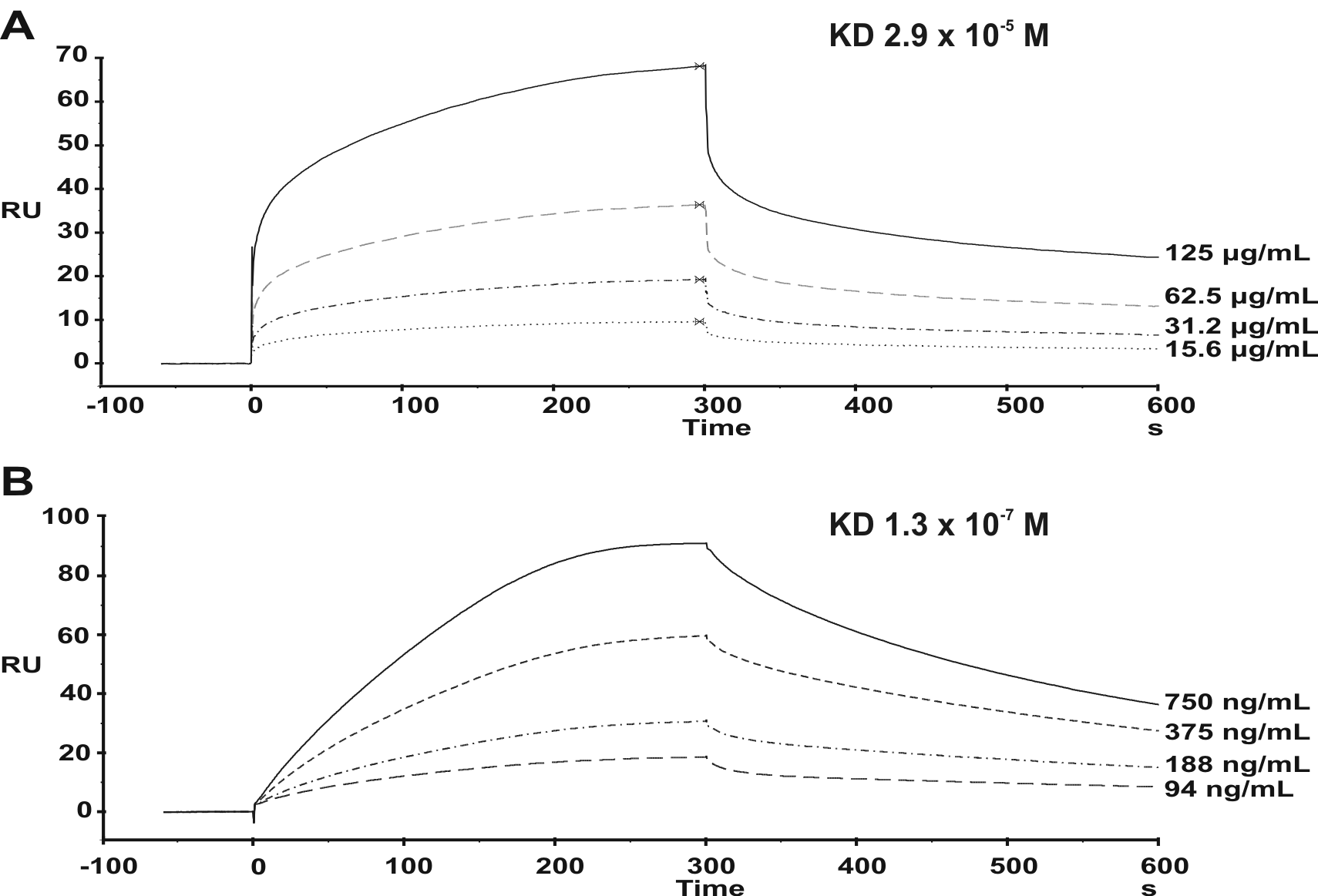

Supplement: FIG S7 [file mBio.01612-20-sf007.tif]

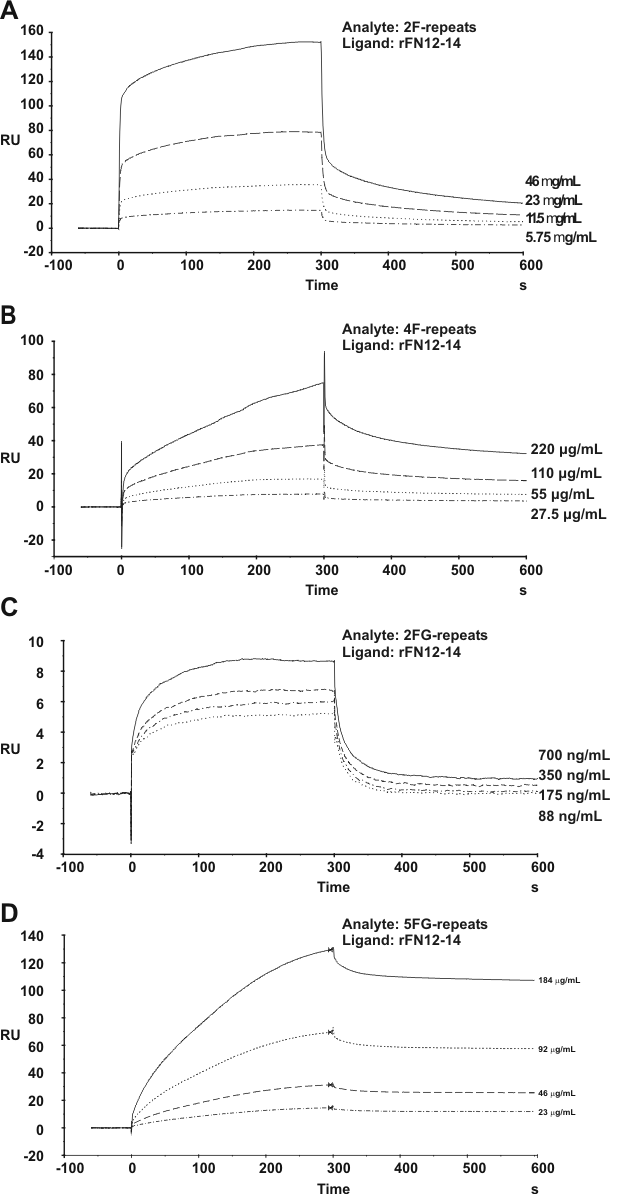

Supplement: FIG S8 [file mBio.01612-20-sf008.tif]

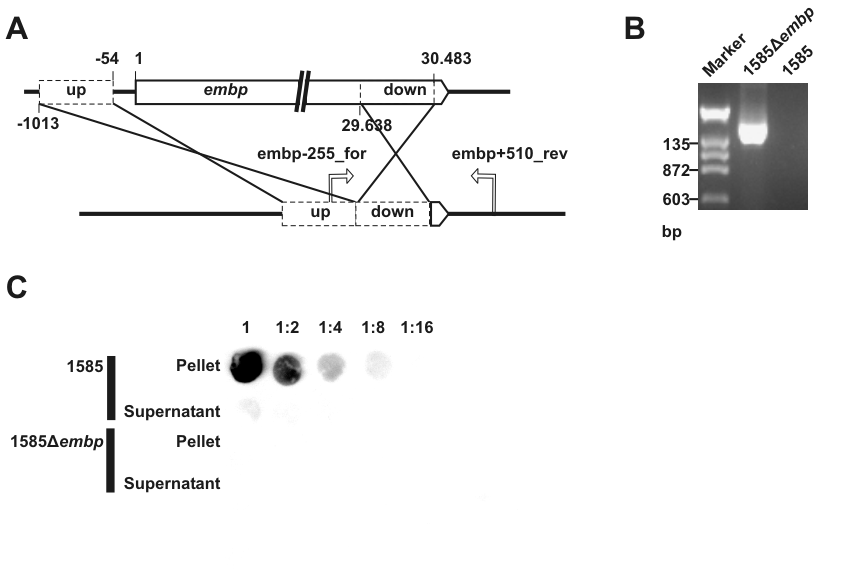

Supplement: FIG S9 [file mBio.01612-20-sf009.tif]
