# Supplementary material for: A Giant Extracellular Matrix Binding Protein of Staphylococcus epidermidis Binds Surface-Immobilized Fibronectin via a Novel Mechanism
Source: mBio. 2020 Oct 20;11(5):e01612-20. doi: 10.1128/mBio.01612-20 (PMC7587433; doi:10.1128/mBio.01612-20)
Supplement: TABLE S1 [file mBio.01612-20-st001.docx]

**Table S1A: Data collection, phasing and refinement statistics**

|  | F-repeat (native) | F-repeat (SeMet) | FG-repeat (native) |
| --- | --- | --- | --- |
| **Data collection** |  |  |  |
| X-ray source | P11, Petra III, DESY | P11, Petra III, DESY | P11, Petra III, DESY |
| Detector | Pilatus 6M | Pilatus 6M | Pilatus 6M |
| Space group | P1 | P1 | P2_1_2_1_2_1_ |
| Cell dimensions |  |  |  |
| *a, b, c* (Å) | 30.80, 34.25, 40.16 | 30.96, 34.24, 40.30 | 25.80, 45.59, 99.66 |
| α, β,γ (º) | 95.9, 103.1, 113.5 | 95.9, 103.1, 113.8 | 90.0, 90.0, 90.0 |
|  |  |  |  |
| Wavelength (Å) | 0.9670 | 0.9795 | 0.9796 |
| Resolution (Å) | 27.00-1.39 (1.44-1.39) | 27.00-2.00 (2.12-2.00) | 26.85-1.55 (1.60-1.55) |
| Total reflections | 91939 (7835) | 33285 (5404) | 198402 (14808) |
| Redundancy | 3.7 (3.6) | 3.5 (3.6) | 11.2 (8.8) |
| Wilson B-factor (Å^2^) | 10.95 | 31.27 | 25.59 |
| *R_meas_* | 0.048 (0.449) | 0.071 (0.232) | 0.040 (0.594) |
| *R*_merge_ | 0.041 (0.384) | 0.061 (0.196) | 0.039 (0.558) |
| CC_1/2_ | 0.999 (0.875) | 0.998 (0.966) | 1 (0.941) |
| *I*/σ*I* | 18.93 (2.96) | 12.95 (4.67) | 31.89 (3.56) |
| Completeness (%) | 86.9 (77.6) | 97.2 (95.8) | 99.1 (95.8) |
|  |  |  |  |
| **Refinement** |  |  |  |
| Reflections used | 24797 (2187) |  | 17659 (1685) |
| Reflection used for R_free_ | 1214 (108) |  | 901 (107) |
| R_work_ | 0.156 (0.233) |  | 0.203 (0.268) |
| R_free_ | 0.186 (0.292) |  | 0.230 (0.280) |
| No. atoms | 1523 |  | 1065 |
| Protein | 1261 |  | 996 |
| Ligand/ion | / |  | / |
| Water | 262 |  | 69 |
| Average B-factor (Å^2^) | 15.40 |  | 31.78 |
| Macromolecules | 13.17 |  | 31.47 |
| Water | 26.14 |  | 36.17 |
| R.m.s deviations |  |  |  |
| Bond lengths (Å) | 0.007 |  | 0.005 |
| Bond angles (º) | 1.10 |  | 0.90 |
| Ramachandran |  |  |  |
| favored (%) | 99.35 |  | 98.39 |
| allowed (%) | 0.65 |  | 1.61 |
| outliers (%) | / |  | / |
| PDB code | 6GV8 |  | 6GV5 |
| *Highest resolution shell is shown in parenthesis. | | | |

Table S1B: SAXS data collection, analysis and modelling

|  | **F-repeats** | **FG-repeats** |
| --- | --- | --- |
| A) Sample details | | |
| Organism | *Staphylococcus epidermidis* | |
| UniProt sequence ID | Q5HPA2 | |
| MW from chemical composition (kDa) | 70.4 | 85.5 |
| Concentration (mg/ml) | 4.1 | 10 |
| Solvent | 50 mM MES, 150 mM NaCl, pH 6 | PBS, 136.5 mM NaCl, 2.65 mM KCl, 8.3 mM Na2HPO4, 2.65 mM KH2PO4, pH 7.4 |
| B) SAXS data-collection parameters | | |
| Instrument/detector | EMBL X33 beam line with a Pilatus 1M-W detector (Blanchet et al. 2012) | EMBL P12 beam line with a Pilatus 2M detector (Blanchet et al. 2015) |
| Wavelength (nm) | 0.15 | 0.124 |
| Camera length (m) | 2.7 | 3.0 |
| *s* measurement range (nm^-1^) | 0.09–6.0 | 0.08–4.5 |
| Normalization | To transmitted intensity by beam-stop counter | |
| Monitoring for radiation damage | Frame-by-frame comparison | |
| Exposure time (s) | 120 | 0.05 |
| Sample temperature (°C) | 10.1 | 23.3 |
| C) Software employed for SAXS data reduction, analysis and interpretation | | |
| SAXS data reduction | SASFLOW (Franke et al. 2012) | |
| Basic analyses: Guinier, p(r), MW | PRIMUSqt from ATSAS 3.0.1 (Franke et al. 2017) | |
| Shape/bead modelling | GASBORi from ATSAS 3.0.1 (Svergun et al. 2001) | |
| Atomic structure modelling | RANCH from ATSAS 3.0.1 (Tria et al. 2015) CRYSOL from ATSAS 3.0.1 (Svergun 1995)  SREFLEX (Panjkovich and Svergun 2016) | |
| D) Structural parameters | | |
| Guinier R_g_ (nm) | 7.0±0.2 | 10.7±0.5 |
| MW from I(0) | 82 | n/a |
| p(r) R_g_ (nm) | 7.7 | 11.4 |
| p(r) D_max_ (nm) | 28 | 40 |
| p(r) χ^2^ | 0.83 | 1.17 |
| MW using the Fischer method (kDa) | 55 | 82 |
| E) Modelling | | |
| GASBOR | | |
| - Number of dummy residues | 632 | 765 |
| - s range for fitting (nm^-1^) | 0.086–5.62 | 0.077–3.75 |
| - Symmetry, anisotropy assumptions | P1, prolate | P1, prolate |
| - χ^2^, CORMAP *p*-values | 1.09, 0.0 | 1.00, 0.08 |
| - Model R_g_, D_max_ (nm) | 8.3, 26.8 | 11.5, 38.8 |
| RANCH/CRYSOL | | |
| - Single repeat structures | PDB entry 6GV8* | PDB entry 6GV5 |
| - s range for fitting (nm^-1^) | 0.092–6.0 | 0.077–4.0 |
| - χ^2^, CORMAP p-values | 2.24, 0.0 | 1.07, 0.17 |
| - Model R_g_, D_max_ (nm) | 6.9, 21.2 | 9.5, 34.8 |
|  | *6GV8 structure was refined with SREFLEX, RMSD=0.57 nm | |
| (f) SASBDB accession codes for data and models | | |
|  | SASDJ92 | SASDJA2 |
